# When Compassion Matters Most: Self-Efficacy as a Moderator of Compassion Effects on Teacher Performance Perceptions

**DOI:** 10.3390/bs16040584

**Published:** 2026-04-14

**Authors:** Ilaria Buonomo, Claudia Russo, Giacomo Angelini, Caterina Fiorilli

**Affiliations:** 1 Department of Human Science, LUMSA University of Rome, 00193 Rome, Italy; i.buonomo1@lumsa.it (I.B.); g.angelini@lumsa.it (G.A.); fiorilli@lumsa.it (C.F.); 2 Department of Health and Life Sciences, Università Europea di Roma, 00163 Rome, Italy

**Keywords:** compassion at work, self-efficacy, teacher performance, resource substitution, moderation, conservation of resources theory

## Abstract

Teacher well-being and performance represent critical challenges for educational systems worldwide. While organizational compassion has been identified as a protective resource, it remains unclear for whom compassion is most beneficial. Drawing on Job Demands–Resources (JD-R) theory and Conservation of Resources (COR) theory, we examined whether teachers’ self-efficacy moderates the relationship between workplace compassion and performance perceptions, testing differential patterns for individual versus organizational performance evaluations. Italian public-school teachers (N = 218; 82% female; M teaching experience = 11.6 years) completed an online survey measuring compassion at work, self-efficacy, and perceptions of individual and organizational performance. We employed a two-stage approach, first validating the measurement model through Confirmatory Factor Analysis (CFA), then testing moderation hypotheses using path analysis with mean-centered variables. Bootstrap confidence intervals (5000 iterations) verified the reliability of interaction effects. Self-efficacy significantly moderated the effect of compassion on individual performance perceptions (β = −0.006, *p* = 0.006; bootstrap 95% CI: [−0.010, −0.002]), revealing a compensatory pattern. Teachers with lower self-efficacy benefited substantially from workplace compassion (simple slope β = 0.31, *p* < 0.001), whereas teachers with high self-efficacy showed no significant benefit (β = 0.06, ns). The hypothesized synergistic effect on organizational performance perceptions was not supported (β = 0.006, *p* = 0.027; bootstrap CI included zero). Organizational compassion functions as a compensatory resource, most powerfully supporting teachers who lack personal resources. This challenges assumptions that organizational interventions uniformly benefit all employees and suggests that compassion-based interventions should be strategically targeted toward teachers experiencing lower self-efficacy. The study advances theoretical understanding of resource substitution mechanisms and provides actionable guidance for optimizing limited organizational resources in educational settings.

## 1. Introduction

Teachers play a pivotal role in shaping student learning and societal outcomes (Russo et al., 2024; Sharma, 2025). A growing body of research emphasizes that teacher well-being is not only vital for teachers’ own health but also for student success and overall school performance (e.g., Y. Li et al., 2025). For instance, teachers who report higher levels of well-being tend to foster better student academic achievement and contribute to a more positive and collaborative school climate (Hine et al., 2022). However, there is substantial evidence that teachers face increasingly complex stressors, such as the increasing complexity of teaching roles, intensifying job demands, and persistent resource constraints. In many countries, including Italy, where the present study was conducted, teachers are expected to deliver high-quality instruction, manage emotional labor and administrative pressures, and meet evolving social expectations (Hascher & Waber, 2021; Marcedula et al., 2026). These conditions are associated with elevated stress and exhaustion, which may undermine instructional quality, professional commitment, and overall effectiveness (Billehøj, 2007). Given this evidence, it is crucial to identify individual and organizational resources that can sustain teachers’ functioning, conceptualized as the result of dynamic interactions between personal, contextual resources, and adaptive processes over time (Mansfield, 2020). Teacher performance represents a key indicator of both individual functioning and school effectiveness, as it captures teachers’ perceived capacity to accomplish their professional tasks and contribute to organizational goals. More specifically, performance perceptions might decline at both the individual and organizational level. Individual performance perception reflects how teachers evaluate their own professional effectiveness, drawing on personal experiences, classroom interactions, and direct feedback (Layek & Koodamara, 2024), whereas organizational performance perception reflects teachers’ assessments of how well their school as a whole is functioning, an evaluation shaped by collective processes and systemic factors that extend beyond any individual teacher’s sphere of influence (Abu-Jarad et al., 2010). Whether individual and organizational resources such as compassion and self-efficacy operate similarly across these two levels is an open question that the present study addresses.

### 1.1. Workplace Compassion as an Organizational Resource

One promising contextual resource within school settings is workplace compassion. Compassion, literally meaning “to suffer together,” reflects not only an empathic awareness of others’ pain but also a genuine motivation to alleviate or prevent future suffering (Anstiss, 2016). In organizational contexts, compassion is expressed when colleagues or leaders recognize a co-worker’s distress, experience empathic concern, and take supportive actions to reduce that suffering (Dutton et al., 2014). In this scenario, workplace compassion may originate from multiple sources, including colleagues, supervisors, and the organization as a whole (Lilius et al., 2008), and it can manifest in behaviors ranging from small acts of kindness and emotional support to formal policies designed to assist employees facing difficulties (Kanov et al., 2004). By signaling a caring climate and fostering a sense of community within the organization (Buonomo et al., 2022), compassion at work represents an important protective factor for employee well-being and performance. Consistent with the Job Demands–Resources (JD-R) model (Bakker & Demerouti, 2007), this collective support can buffer the impact of stressors by reducing employees’ sense of isolation, encouraging help-seeking, and facilitating recovery from setbacks. Empirical evidence shows that receiving compassion in the workplace is associated with significant reductions in burnout (Eldor, 2018; Román-Calderón et al., 2022). For example, in healthcare settings, employees who experience greater compassion from colleagues report lower emotional exhaustion and depersonalization, alongside a stronger sense of professional accomplishment (Figley, 2002; Lombardo & Eyre, 2011). These benefits are likely to extend to educational contexts, where teaching is similarly relational and emotionally demanding, making compassion among co-workers a potentially crucial protective resource (Buonomo et al., 2022). Consistently, Eldor and Shoshani (2016) found that, among Israeli teachers, compassion received from colleagues and school principals was positively associated with emotional vigor, organizational commitment, and job satisfaction, and negatively associated with burnout, with these relationships being mediated by positive affect. Moreover, Matos et al. (2022) showed that an 8-week compassion training program significantly increased levels of compassion, self-compassion, positive affect, and well-being, while reducing anxiety, stress, and burnout compared to the control group. Even when not explicitly framed as compassion, forms of organizational social support have consistently been linked to stress reduction in teachers. Support from colleagues and administrators functions as a key coping resource that prevents job demands from developing into burnout (Bakker et al., 2005). A compassionate school culture may also foster positive outcomes such as teacher engagement, commitment, and performance. Indeed, prior research found a significant positive relationship between workplace compassion and performance indicators (Aboul-Ela, 2017). Despite this promising evidence, however, few studies have been conducted to understand the conditions under which workplace compassion leads to such positive outcomes. Moreover, considering the Conservation of Resources (COR) theory (Hobfoll, 1989), individuals are motivated to acquire, retain, and protect resources, both material and psychological, and they react strongly to the potential or actual loss of these resources. In this regard, integrating the JD-R model and COR theory provides a useful framework to understand how organizational resources, such as workplace compassion, may interact with individual resources in shaping work-related outcomes. While the JD-R model emphasizes the role of resources in sustaining performance and well-being, COR theory further suggests that resources may operate either synergistically or compensatorily, depending on individuals’ existing resource levels. Thus, workplace compassion can serve as both a supportive condition and a compensatory mechanism. When personal resources are depleted, contextual resources such as compassion can help to replenish or replace them. Based on this rationale, further investigation is particularly needed to clarify how organizational resources, such as compassion at work, interact with individual personal resources in shaping teachers’ functioning indicators, such as performance.

### 1.2. Self-Efficacy as an Individual Resource

While a compassionate environment is one aspect of the equation, teachers may also bring personal resources that could help them cope with demands. A core personal resource in this context is self-efficacy, namely the individual’s belief in their own ability to effectively organize and execute the actions needed to reach specific goals (Bandura, 1997; Tschannen-Moran et al., 1998). Self-efficacy is rooted in Bandura’s social cognitive theory, which emphasizes human agency and the idea that individuals can control their behavior and environment through their beliefs (Bandura, 1986). Accordingly, individuals with high levels of general self-efficacy are more likely to view challenging tasks as opportunities for growth rather than as threats to be avoided (Bandura, 2006). In the current educational landscape, the role of a teacher encompasses a broad spectrum of responsibilities, ranging from classroom instruction to project coordination, collaboration with colleagues, administrative duties, and regular interaction with school administrators (Marcedula et al., 2026). In this complex and dynamic professional landscape, teachers must continually adapt to conflicting demands and unpredictable challenges. In this sense, a broader form of professional self-efficacy is particularly important, reflecting teachers’ confidence in their ability to draw on personal and social resources and manage diverse work situations effectively.

Consistently, in the teaching context, previous evidence highlighted that self-efficacy is a key individual factor in promoting teacher performance, job satisfaction and higher levels of well-being (Tschannen-Moran & Hoy, 2001), preventing the risk of developing burnout (S. Li, 2023). Moreover, self-efficacy is positively associated with teachers’ professional identity, conceptualized as the individuals’ understanding, perception, and sense of belonging to their professional role, encompassing values, beliefs, and emotional commitment to teaching (Beijaard et al., 2004). Indeed, previous studies showed that self-efficacy may contribute to the development of professional identity by shaping how teachers interpret their capabilities, engage with professional challenges, and construct meaning around their professional role (Canrinus et al., 2012; Matsuda & Hamada, 2026; Sun et al., 2025). Because self-efficacy contributes to the development of professional identity by shaping how teachers interpret their capabilities and construct meaning around their professional role, teachers with lower self-efficacy may also hold a less consolidated professional sense of self, making them more reliant on contextual signals of recognition and support. This suggests that self-efficacy may influence not only teachers’ confidence in their capabilities but also the degree to which they look to the social environment when forming judgments about their own professional effectiveness, which makes it a theoretically grounded moderator of the relationship between workplace compassion and performance perceptions.

However, personal resources do not operate in isolation but rather interact with the resources present in the context (Bandura, 1982; Jensen et al., 2025; Xanthopoulou et al., 2007). Emerging evidence suggests that a combination of strong personal and supportive environmental resources yields the best outcomes (Granziera et al., 2021; Johari et al., 2022; Xanthopoulou et al., 2007). For example, a recent study revealed that social support and self-efficacy significantly predict the quality of teachers’ working lives, with greater support and self-efficacy being linked to improved well-being and productivity (Jaguaco et al., 2022). These findings suggest that teachers who perceive themselves as efficacious and supported by colleagues are more likely to experience positive work-related outcomes. Conversely, in line with COR theory, teachers with lower levels of self-efficacy may particularly benefit from a compassionate and supportive work environment, as contextual resources can compensate for or replenish limited personal resources (Hobfoll et al., 2018). In practice, even less confident teachers can function effectively when they are part of a supportive and collaborative professional environment.

### 1.3. The Present Study

Despite growing evidence on the positive role of workplace compassion and self-efficacy in educational settings, few studies have examined how these factors interact to influence teachers’ performance. In particular, there is a lack of research into whether organizational resources, such as workplace compassion, compensate for or amplify individual resources, such as self-efficacy.

Based on this rationale, the present study examines how both organizational (i.e., workplace compassion) and personal (i.e., teacher self-efficacy) resources jointly relate to teacher performance, conceptualized at both the individual and organizational level. Consistent with COR theory, we explore whether these resources have additive or interactive effects. Specifically, we examined whether workplace compassion primarily serves a compensatory function, thus being particularly beneficial for teachers with lower self-efficacy, or an amplification function, enhancing performance especially among highly self-efficacious teachers.

Based on the evidence highlighted above, we hypothesized that:

**H1.**  *Workplace compassion will be associated with higher levels of both individual and organizational performance.*

**H2.**  *Self-efficacy will be associated with higher levels of both individual and organizational performance.*

**H3.**  *Self-efficacy will significantly interact with workplace compassion. Nonetheless, due to the scarcity of prior empirical research clarifying the direction and functional form of this interaction, and the broader uncertainty concerning how personal resources may influence the impact of organizational resources on performance, we adopted a non-directional, exploratory approach.*

## 2. Materials and Methods

### 2.1. Participants and Procedure

A total of 223 Italian public-school teachers participated in an online survey. Missing data were minimal (<3% per variable) and were handled through listwise deletion, resulting in a final analytic sample of N = 218, on which all subsequent analyses were conducted. Analyses were conducted using Mplus 8 (Muthén & Muthén, 1998–2017). This sample was predominantly female (82%), with 18% male, and represented all levels of public education: secondary schools (57%), primary schools (27%), and preschools (16%). Teaching experience ranged from 1 to 39 years (M = 11.6 years, SD = 9.4), with a median of 10 years, indicating a sample with diverse career stages from novice to veteran educators. Most participants held permanent teaching contracts (76%), with 24% on temporary contracts. 

Participants were recruited through snowballing sampling procedures. The survey was administered online via Google Forms between February and May 2024. Participation was voluntary and anonymous. Ethical approval was obtained from the LUMSA University Ethical Board. All participants provided informed consent before beginning the survey.

### 2.2. Measures

#### 2.2.1. Perceived Organizational Performance

Perceived Organizational Performance (6 items; α = 0.95) was measured through a scale adapted from Salminen et al. (2017) (sample item: “I am satisfied with the relations between management and teaching staff”). Response scale ranged from 1 (Strongly disagree) to 5 (Strongly agree).

#### 2.2.2. Perceived Individual Performance

Perceived Individual Performance (3 items; α = 0.77) was measured through a scale adapted from Salminen et al. (2017), a measure widely adopted for the assessment of different levels of perceived performance (see, for example, Buonomo et al., 2022) (sample item: “I am satisfied with my work performance”). Response scale ranged from 1 (Strongly disagree) to 5 (Strongly agree).

#### 2.2.3. Compassion at Work

Compassion at Work (3 items; α = 0.87) was measured through the Compassion at work scale by Lilius et al. (2008). The scale has been widely used across different organizational contexts and professional groups, demonstrating good reliability and adaptability (see, for example, Hu et al., 2018; Zoghbi-Manrique-de-Lara et al., 2020). A sample item is: “I receive compassion from my co-workers”. Response scale ranged from 1 (Never) to 5 (Always).

#### 2.2.4. Self-Efficacy

Self-Efficacy (6 items; α = 0.88) was measured through the COPSOQ III (Burr et al., 2019) subscale (sample item: “How well does this description fit you as a person? I feel confident that I can handle unexpected events”), using a scale from 0 (Does not fit) to 100 (Fits perfectly). Although domain-specific measures of teaching efficacy exist (e.g., Tschannen-Moran & Hoy, 2001), we opted for a general professional self-efficacy measure for two reasons. First, the contemporary teaching role extends well beyond classroom instruction to encompass project coordination, interdisciplinary collaboration, administrative tasks, and engagement with school leadership (Marcedula et al., 2026), making a broader measure of professional confidence arguably more representative of the full range of demands teachers face. Second, Bandura (1997) distinguished between task-specific and generalized efficacy beliefs, noting that the latter reflect individuals’ confidence in mobilizing resources and managing unexpected professional demands across varied situations, which is the construct captured by the COPSOQ III subscale used here.

### 2.3. Data Analysis Strategy

We employed a two-stage analytic approach (Anderson & Gerbing, 1988) to test our moderation hypotheses. In the first stage, we conducted confirmatory factor analysis (CFA) to validate the measurement model and ensure adequate psychometric properties of all constructs. Confirmatory factor analysis (CFA) is a theory-driven statistical technique used to test whether observed indicators (e.g., Likert-scale items) adequately represent the underlying latent constructs. It allows researchers to assess the adequacy of the measurement model and the reliability and validity of the constructs (Brown & Moore, 2012). To reduce model complexity while preserving construct validity, we used parceling for multi-item scales (Little et al., 2002). Parcels were created by averaging items within each construct, with a random assignment of items to parcels. For scales with fewer than four items, individual items were retained as indicators.

The CFA demonstrated excellent fit and strong factor loadings across all constructs (range: 0.70–0.95, M = 0.85), supporting convergent validity. Specifically, parceling was optimized to maximize factor loadings while maintaining conceptual coherence, yielding the following structure: Organizational Performance (3 parcels of 2 items each), Individual Performance (3 individual items), Compassion at Work (3 individual items) and Self-Efficacy (3 parcels of 2 items each). All standardized factor loadings exceeded 0.70, substantially above conventional thresholds (Hair et al., 2010).

In the second stage, we extracted factor scores from the CFA and used these composite scores in path analyses to test structural hypotheses. Moderation effects were estimated using product terms of mean-centered predictors (Aiken et al., 1991). We used maximum likelihood estimation with robust standard errors (MLR) to account for potential non-normality. To verify the reliability of interaction effects, we computed bootstrap confidence intervals with 5000 iterations at the 95% confidence level.

## 3. Results

### 3.1. Descriptive Statistics and Correlations

Table 1 presents means, standard deviations, reliabilities, and zero-order correlations among study variables. All reliability coefficients exceeded 0.70 (range: 0.73–0.96), supporting internal consistency. Compassion at work correlated positively with organizational performance perceptions (r = 0.59, *p* < 0.001) and individual performance perceptions (r = 0.23, *p* < 0.01). Self-efficacy correlated positively with individual performance (r = 0.31, *p* < 0.001) but showed a non-significant correlation with compassion (r = −0.05, ns), suggesting that these represent distinct resource domains as hypothesized. These preliminary correlations provide initial support for examining self-efficacy as a moderator of compassion effects.

### 3.2. Common Method Variance Assessment

Given the cross-sectional self-report design, we assessed potential common method variance using Harman’s single-factor test (Podsakoff et al., 2003). When all items were entered into an exploratory factor analysis, the first unrotated factor accounted for 32% of the total variance, well below the 50% threshold that would indicate substantial common method bias. This suggests that common method variance is not a major concern in the present study.

### 3.3. Hypothesis Testing: Moderation Effects

**Figure 1:**
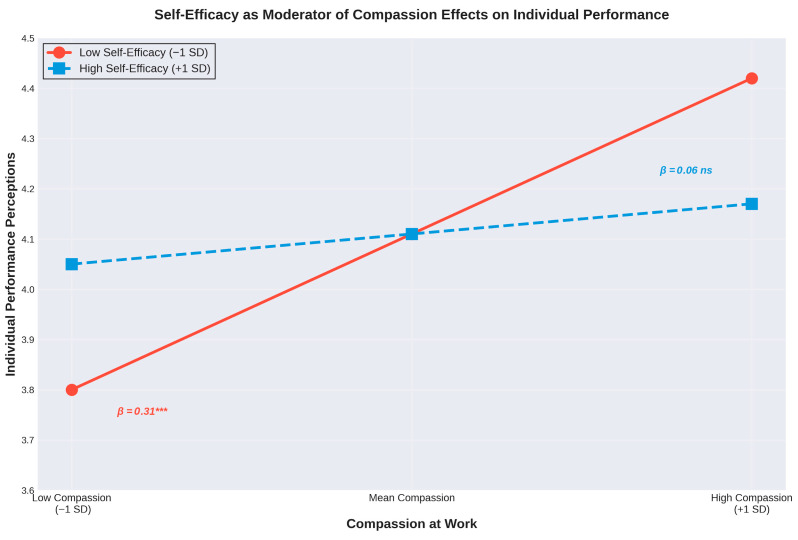
Interaction between compassion at work and self-efficacy in predicting individual performance perceptions. Note. SD from the mean. The compensatory pattern shows that compassion most strongly benefits teachers with lower self-efficacy (β = 0.31, *** *p* < 0.001), with diminishing returns as self-efficacy increases (high self-efficacy: β = 0.06, ns).

We tested whether self-efficacy moderated the relationship between compassion at work and performance perceptions. Following established recommendations for testing interactions (Aiken et al., 1991), we mean-centered predictor variables before creating product terms. Results are presented in Table 2 and Figure 1.

### 3.4. Organizational Performance Perceptions (Hypothesis 1)

We hypothesized that self-efficacy would synergistically enhance the positive relationship between compassion and organizational performance perceptions. The main effects model (without interaction) showed that both compassion (β = 0.525, *p* < 0.001) and self-efficacy (β = 0.135, *p* = 0.016) were positively associated with organizational performance. When the interaction term was added, the analytic *p*-value was 0.027 (β = 0.006, SE = 0.003); however, the bootstrap 95% CI ([−0.001, 0.011]) included zero. Given this divergence, and consistent with recommendations for interaction testing in structural models (Hayes, 2018; Preacher & Hayes, 2008), we rely on the bootstrap CI as the primary inferential criterion and conclude that the evidence for synergistic moderation on organizational performance is insufficient. Hypothesis 1 was not supported.

The full model (including the interaction term) explained substantial variance in organizational performance perceptions (R^2^ = 0.389, *p* < 0.001), indicating that compassion and self-efficacy together account for approximately 39% of the variance, even without confirmed moderation effects.

### 3.5. Individual Performance Perceptions (Hypothesis 2)

We hypothesized that self-efficacy would moderate the relationship between compassion and individual performance perceptions. The main effects model showed that both compassion (β = 0.185, *p* < 0.001) and self-efficacy (β = 0.311, *p* < 0.001) showed a positive association with individual performance. Critically, the interaction term was significant and negative (β = −0.006, *p* = 0.006; bootstrap 95% CI: [−0.010, −0.002]), indicating that self-efficacy moderated the effect of compassion on individual performance evaluations. Hypothesis 2 was supported.

To interpret this interaction, we conducted simple slopes analyses at ±1 SD of self-efficacy (Aiken et al., 1991). As illustrated in Figure 1, the pattern revealed a compensatory mechanism:

- Low Self-Efficacy (−1 SD): The effect of compassion was strong and positive (β = 0.312, *p* < 0.001), indicating that teachers with lower self-efficacy benefited substantially from workplace compassion.
- Mean Self-Efficacy: The effect of compassion remained positive and significant (β = 0.185, *p* < 0.001).
- High Self-Efficacy (+1 SD): The effect of compassion was reduced to non-significance (β = 0.059, *p* = 0.258), indicating that teachers with high self-efficacy did not require additional external resources from workplace compassion.

This compensatory pattern suggests that workplace compassion functions as an effective substitute for personal resources: when teachers lack self-efficacy, compassion provides critical support for their individual performance evaluations. However, when teachers already possess strong self-efficacy beliefs, the additional benefit of compassion becomes negligible.

The full model explained significant variance in individual performance perceptions (R^2^ = 0.187, *p* < 0.001), with compassion, self-efficacy, and their interaction accounting for approximately 19% of the variance.

### 3.6. Summary of Findings

Our analyses revealed a compensatory moderation pattern for individual performance perceptions. Self-efficacy significantly moderated the relationship between compassion at work and individual performance evaluations (β = −0.006, *p* = 0.006), such that compassion was most beneficial for teachers reporting lower self-efficacy. Simple slope analyses confirmed that the positive effect of compassion diminished as self-efficacy increased, becoming non-significant at high levels of self-efficacy. This pattern demonstrates that workplace compassion serves as an effective substitute for personal resources in supporting individual performance perceptions.

In contrast, the hypothesized synergistic moderation for organizational performance perceptions was not supported. While both compassion and self-efficacy showed linear positive relationships with organizational performance, the interaction between these variables did not reach robust statistical significance. This differential pattern across performance types suggests that resource dynamics may operate differently depending on whether teachers evaluate their individual contributions versus their school’s collective effectiveness.

## 4. Discussion

This study tested whether and how self-efficacy moderates the effects of workplace compassion on teachers’ perceived individual and organizational performance. Drawing on the COR theory (Hobfoll, 2002), we examined if workplace compassion (as an organizational resource) would amplify the benefits of personal resources (synergistic pattern) or compensate for their absence (substitution pattern). The results reveal a compensatory pattern: receiving compassion most strongly benefits teachers with lower self-efficacy in individual performance self-evaluations, while producing no significant benefit for high self-efficacy teachers. This finding suggests that organizational resources, such as compassion, may function as substitution mechanisms rather than amplification of personal resources.

### 4.1. Main Effects of Compassion and Self-Efficacy on Performance Perceptions

Before examining the moderating role of self-efficacy, we consider the main effects of both received compassion at work and self-efficacy on performance perceptions. Both variables showed significant positive relationships with perceived performance, both at the individual and at the organizational level. In other words, teachers who perceived greater compassion in their workplace and those reporting higher self-efficacy tended to evaluate both their own teaching effectiveness and their school’s overall performance more positively. These main effects align with the COR theory (Hobfoll, 2002), which posits that both personal resources (i.e., self-efficacy) and organizational resources (i.e., workplace compassion) support valued outcomes (in our case, performance). The positive association between compassion and performance perceptions is consistent with research documenting compassion’s role in fostering psychological safety, reducing stress, and enhancing well-being (Dutton et al., 2014). Similarly, the self-efficacy–performance link reflects decades of research showing that confidence in one’s capabilities is positively associated with actual and perceived performance across diverse domains (Bandura, 1997).

These main effects are worth situating within the specific context of the teaching profession. Teaching is an emotionally demanding occupation in which practitioners are exposed to continuous relational requests from students, parents, and colleagues, while often working in conditions of professional isolation (Lortie, 1975; Hargreaves, 1994). In such a context, compassion among colleagues may carry particular weight as a resource, because it addresses a structural deficit: teachers who spend most of their working day in the relative solitude of their classrooms have fewer routine opportunities to receive socio-emotional support from peers. Consistently, Eldor and Shoshani (2016) found that compassion within the school staff predicted teacher work engagement, suggesting that, when collegial compassion occurs in school settings, its effects on professional outcomes are substantial.

The Broaden-and-Build theory (Fredrickson, 1998, 2001) offers a complementary approach for understanding these main effects, with particular regard to the role of received compassion. According to Fredrickson, positive emotions (including feeling valued, supported, and cared for) broaden individuals’ momentary thought–action repertoires and, over time, build enduring personal resources including resilience, social bonds, and psychological capital. In the context of our findings, teachers who perceive compassion in their workplace may experience positive emotional states that progressively build their sense of professional competence and their appreciation of both individual and organizational functioning. Empirical support for this mechanism comes from Choi and Ko (2024), who showed that experienced compassion at work predicted job performance through a serial pathway involving positive emotions and proactive job crafting behaviors, a sequence consistent with the building component of Fredrickson’s theory. This building mechanism also resonates with the resource gain spirals posited by COR theory (Halbesleben et al., 2014) and incorporated into the JD-R framework as applied to teachers (Granziera et al., 2021): the positive emotions elicited by compassion accumulate into durable resources, which, in turn, foster further positive appraisals of performance.

From this combined COR–Broaden-and-Build perspective, an additive or synergistic pattern would predict that teachers with high self-efficacy benefit most from compassionate work environments, as their already robust personal resources would catalyze stronger gain spirals, creating a “rich get richer” dynamic. However, as we discuss below, the interaction effects tell a different and more nuanced story.

### 4.2. The Compensatory Pattern: Why Compassion Benefits Lower Self-Efficacy Teachers Most

Our findings reveal a compensatory gradient pattern in how workplace compassion relates to individual performance perceptions, with benefits strongest for teachers with lower self-efficacy, moderate for those with average self-efficacy, and negligible for high self-efficacy teachers. This pattern supports resource substitution mechanisms within the Conservation of Resources theory (Hobfoll, 2002), so that organizational resources prove most valuable where personal resources are lacking or moderate. However, two theories suggest psychosocial mechanisms that may underlie the moderation paths observed in this study.

The Crossover theory (Hobfoll et al., 2018) suggests that the emotional tone of a compassionate environment can be experienced by individuals. For teachers with low self-efficacy, this experienced compassion may provide a powerful external counterweight to their doubts about the degree of control and effectiveness at work, making them more likely to weigh the external signals about their work when forming performance self-perceptions. For teachers already confident in their abilities, the crossover effect is more an alignment between the external compassionate feedback and their internal state, thus adding less incremental value to an already-positive self-perception.

The Broaden-and-Build theory (Fredrickson, 1998, 2001) may further illuminate this shift, although with an important theoretical qualification. As discussed above, the theory was originally formulated to explain how positive emotions broaden cognitive and behavioral repertoires and progressively build resources. While this mechanism aligns more naturally with synergistic patterns of resource accumulation, we propose that an extension of the broadening component can help account for the compensatory pattern observed here. The broadening effect of positive emotions may operate across all levels of self-efficacy, but its marginal impact is likely to differ as a function of individuals’ baseline repertoires. Teachers with lower self-efficacy tend to operate with narrower cognitive and behavioral repertoires and are characterized by greater rigidity in problem-solving, heightened sensitivity to threat, and more restricted self-evaluation frameworks (Bandura, 1997). Thus, they could benefit more from the broadening effect of compassionate environments, because they have more room for their repertoires to expand. For these teachers, the positive emotions elicited by compassion may open up new ways of appraising their own effectiveness: noticing aspects of their teaching they had overlooked, reframing challenges as manageable rather than threatening, and arriving at more balanced self-evaluations. Recent evidence from the Italian school context confirms that self-efficacy is not a fixed trait but a dynamic psychological state that responds to positive experiences in the work environment (Angelini et al., 2025). This responsiveness supports the idea that teachers with lower self-efficacy may respond more strongly to environmental signals of recognition and support, as their lower confidence may make them more receptive to external, positive input.

Conversely, for teachers with high self-efficacy, whose repertoires are already broad and well-functioning, the same broadening mechanism may yield lower returns, a sort of “ceiling effect”, in which additional positive emotions produce limited incremental change in already expansive self-appraisals. While our cross-sectional data do not allow us to test this diminishing marginal returns mechanism, the observed gradient pattern (i.e., strongest effects at low self-efficacy, moderate at average, negligible at high) is consistent with this interpretation.

### 4.3. Differentiated Functions of Compassion in the Teaching Profession

The compensatory pattern described above raises a broader question about what compassion does, and for whom, in the specific context of teaching. We address this question by considering three interrelated aspects: the particular vulnerability that low self-efficacy creates in this profession, the possibility that compassion serves different functions at different resource levels, and the divergence between individual and organizational performance perceptions.

Teaching involves sustained emotional labor: managing classroom dynamics, responding to students’ needs, navigating relationships with parents and administrators. Teachers with lower self-efficacy face these demands with fewer internal resources to draw upon, which makes them more susceptible to stress, less persistent when encountering difficulties, and at greater risk of emotional exhaustion (Bandura, 1997; Schwarzer & Hallum, 2008). A defining feature of low self-efficacy, however, is not only resource scarcity but also a particular sensitivity to external signals about one’s own competence. Teachers with lower professional confidence tend to operate with restricted self-evaluative frameworks, in which negative information is weighted more heavily than positive, and challenges are more readily interpreted as evidence of inadequacy (Bandura, 1997). In this context, the mere perception of receiving compassion from colleagues and supervisors may carry disproportionate weight: it signals that others in the work environment notice their difficulties and respond with care, which is precisely the kind of external input that can interrupt restricted self-evaluative patterns. This responsiveness to external input may also reflect the relationship between self-efficacy and professional identity described above. Because self-efficacy shapes how teachers interpret their capabilities and construct meaning around their professional role (Canrinus et al., 2012; Sun et al., 2025), teachers with lower self-efficacy may hold a less consolidated professional sense of self, and may therefore be more sensitive to signals from the work environment as a source of external grounding for their self-perceptions as professionals. From this perspective, the perception of receiving compassion from colleagues may carry particular weight not only as emotional support, but as a signal that one’s professional efforts are noticed and valued within the school community. Notably, this effect emerges in our data with a highly parsimonious measure of received compassion (Lilius et al., 2008), one that captures the general perception of being the recipient of compassion without specifying its form or structure. The fact that the compensatory moderation pattern is detectable even at this level of measurement suggests that the threshold for activation is relatively low: it is the perception of being seen and cared for, rather than any specific or elaborated form of compassionate intervention, that appears to matter most for teachers whose professional self-concept is most in need of external anchoring.

At the same time, the absence of a significant moderation effect for high self-efficacy teachers should not be interpreted as evidence that compassion is irrelevant for this group. A more careful reading of the data, informed by COR theory’s distinction between resource gain and resource loss prevention (Hobfoll, 2002), suggests that compassion may serve a different function depending on the recipient’s resource endowment. For teachers with lower self-efficacy, compassion appears to operate as a transformative resource: it shifts their performance self-evaluations upward by broadening restricted repertoires, an effect that is not observed for their high-efficacy colleagues. For teachers whose self-efficacy is already high, the same compassionate environment may serve a conservative or maintenance function, one that does not produce visible gains in performance self-perceptions (because these are already near their ceiling) but may protect existing resources from erosion under conditions of sustained demand. This interpretation is consistent with Hobfoll’s (2002) principle that resource loss is more psychologically impactful than resource gain: a compassionate workplace may prevent losses that would otherwise occur, even if this preventive effect does not manifest as improved self-evaluations in cross-sectional data. It is also possible that, for high self-efficacy teachers, compassion produces its effects on outcomes other than performance perceptions, such as job satisfaction, affective commitment, or intention to remain in the profession, a possibility that future research could address by examining moderation patterns across a broader set of outcomes.

In sum, the compensatory pattern observed here does not imply that compassionate work environments are superfluous for well-resourced professionals. Rather, it suggests that the same organizational resource may play qualitatively different roles, from transformation to stabilization, depending on where the recipient’s resource system has the most room to move. For those with depleted personal resources, compassion activates change; for those with adequate resources, it sustains functioning.

This differentiated logic also helps make sense of the divergence between individual and organizational performance perceptions. The compensatory moderation pattern emerged for individual performance but not for organizational performance. Individual performance represents a teacher’s evaluation of their own teaching effectiveness, an assessment rooted in personal experiences, classroom interactions, and direct feedback from students. This domain remains largely within the teacher’s perceived control and is tied to their professional self-concept. In contrast, organizational performance represents the school’s collective effectiveness, an assessment influenced by factors beyond any individual teacher’s sphere of influence, including administrative decisions, resource availability, and systemic constraints. In this regard, Buonomo et al. (2022) found that the relationship between received compassion and teachers’ well-being operated through perceptions of collective school performance rather than through individual self-referential processes, suggesting that organizational-level appraisals may respond to compassion more uniformly across teachers. By contrast, the relationship between compassion and individual performance perceptions may be more psychologically contingent, shaped by self-referential cognitive and emotional processes that operate differently depending on personal resource levels.

The professional isolation that characterizes teaching (Hargreaves, 1994; Lortie, 1975;) may further contribute to this divergence. Teachers who operate as solo practitioners within their classrooms may have a weaker psychological connection to organizational outcomes, making personal resources like self-efficacy less relevant for shaping organizational performance perceptions. When teachers view themselves as individual professionals rather than integral parts of a collective enterprise, the interplay between personal confidence and received compassion may matter less for how they evaluate the school’s effectiveness.

### 4.4. Limitations

Several limitations should be considered when interpreting the present findings.

First, the cross-sectional design precludes causal inference and does not allow conclusions about temporal ordering; thus, all associations should be interpreted as concurrent relationships. Future research should employ longitudinal designs to examine whether workplace compassion prospectively predicts changes in performance perceptions and whether self-efficacy evolves over time in response to compassionate experiences. Second, all study variables were assessed via self-report measures, including perceived performance. This is particularly relevant in teacher research, where self-efficacy has been shown to relate more strongly to self-reported than externally evaluated performance (Jerrim et al., 2025). Although common method variance was examined, Harman’s single-factor test alone does not rule out shared method bias or social desirability effects, which may inflate associations. Third, some measurement issues should be considered. Key constructs were assessed using short scales, which may not fully capture their multidimensional nature. In addition, the use of different response formats (e.g., 0–100 for self-efficacy vs. 1–5 Likert scales for other variables) may affect comparability and parameter estimates, particularly in interaction analyses, despite mean-centering procedures.

Finally, the use of non-probabilistic snowball sampling may limit representativeness and introduce selection biases. In addition, the gender imbalance in the sample, although broadly consistent with the Italian teaching workforce (OECD, 2018), may further constrain generalizability to more diverse populations.

## 5. Practical Implications

The present findings suggest that compassion at work may function as a contextually relevant organizational resource. In practical terms, schools may derive meaningful, albeit potentially modest, performance-related benefits by prioritizing compassion-oriented practices for teachers with lower self-efficacy, for whom compassionate experiences appear to strengthen more favorable appraisals of their own effectiveness (Hobfoll, 2002). This has direct implications for how limited support resources are allocated. For instance, school leaders could identify teachers in need of additional support through routine professional development needs assessments, structured mentoring conversations, or early-career induction processes, and then direct resources toward those most likely to benefit most from additional support.

Importantly, these implications do not necessarily require resource-intensive programs: it is the felt sense of being seen and cared for by colleagues and supervisors, rather than elaborate interventions, that may be particularly consequential (Dutton et al., 2014). Accordingly, schools could implement low-intensity, scalable routines embedded in everyday practice, such as brief peer check-ins, structured collegial debriefing after challenging events, mentoring or coaching for early-career and struggling teachers, and leadership behaviors that normalize help-seeking and communication recognition. At the same time, the absence of a clear moderation effect among high-self-efficacy teachers should not be interpreted as grounds for withholding support from this group. A broadly compassionate school culture remains a worthwhile organizational investment, likely serving a protective and stabilizing function under sustained demands and contributing to more positive evaluations of school functioning overall. Finally, given that self-efficacy may be responsive to contextual experiences, schools may further enhance impact by coupling compassion-based practices with targeted professional development aimed at strengthening efficacy beliefs (e.g., mastery experiences, coaching, structured peer observation), thereby addressing organizational and individual resources in a complementary manner (see also Rehman et al., 2025).

## 6. Conclusions

These findings are consistent with a view of workplace compassion as a compensatory organizational resource rather than an amplifier of existing personal strengths (Hobfoll, 2002). The observed pattern aligns with the resource substitution logic, suggesting that the same contextual resource may play different roles depending on recipients’ baseline resources, with potentially greater relevance where personal resources are constrained and more limited incremental value where they are already strong. The divergence between individual and organizational performance perceptions further suggests that resource dynamics may vary as a function of whether performance is evaluated through a self-referential versus a collectively oriented lens, a distinction that warrants closer attention in future research.

Beyond extending resource-based approaches to the educational context, the study points to the importance of moving beyond uniform assumptions about organizational interventions. In contexts where support resources are limited, compassion-oriented actions may be particularly relevant for teachers with lower self-efficacy, although the magnitude of these effects appears modest and should be interpreted cautiously given the cross-sectional, self-report nature of the data and the focus on perceived performance outcomes. Taken together, these findings offer a preliminary basis for considering how contextual and personal resources may jointly shape teachers’ performance perceptions in school settings.

## Figures and Tables

**Table 1:** Descriptive statistics, reliabilities, and correlations.

Variable	M	SD	α	1	2	3
1. Organizational Performance	3.47	0.91	0.95	—		
2. Individual Performance	4.17	0.71	0.77	0.29 ***	—	
3. Compassion at Work	3.47	1.02	0.87	0.59 ***	0.23 ***	—
4. Self-Efficacy	62.67	19.61	0.88	0.13	0.31 ***	−0.05

Note. *** *p* < 0.001.

**Table 2:** Path analysis results for moderation models.

Model	β	SE	t	*p*	95% CI
Model 1: Organizational Performance					
Compassion at Work	0.525 ***	0.048	10.91	<0.001	[0.431, 0.619]
Self-Efficacy	0.135 *	0.056	2.41	0.016	[0.025, 0.245]
CAW × Self-Efficacy	0.006	0.003	2.23	0.027	[−0.001, 0.011]
R^2^	0.389 ***				
Model 2: Individual Performance					
Compassion at Work	0.185 ***	0.043	4.28	<0.001	[0.100, 0.270]
Self-Efficacy	0.311 ***	0.062	5.02	<0.001	[0.189, 0.433]
CAW × Self-Efficacy	−0.006 **	0.002	−2.76	0.006	[−0.010, −0.002]
R^2^	0.187 ***				

Note. N = 218. Standardized coefficients (β) reported. Bootstrap standard errors (5000 iterations) and 95% confidence intervals based on bias-corrected percentile method. CAW = Compassion at Work. * *p* < 0.05. ** *p* < 0.01. *** *p* < 0.001.

## Data Availability

The data presented in this study are available from the corresponding author upon reasonable request.
